# Parasitic contamination of fresh vegetables sold at central markets in Khartoum state, Sudan

**DOI:** 10.1186/s12941-016-0133-5

**Published:** 2016-03-11

**Authors:** Mona Ali Mohamed, Emmanuel Edwar Siddig, Arwa Hassan Elaagip, Ali Mahmoud Mohammed Edris, Awad Ahmed Nasr

**Affiliations:** Department of Parasitology and Medical Entomology, Faculty of Medical Laboratory Sciences, University of Khartoum, Khartoum, Sudan; Nile College, Khartoum, Sudan; Department of Histopathology and Cytology, Faculty of Medical Laboratory Sciences, University of Khartoum, Khartoum, Sudan

**Keywords:** Parasitic contamination, Fresh vegetables, Intestinal parasites, Khartoum, Sudan

## Abstract

**Background:**

Fresh vegetables are considered as vital nutrients of a healthy diet as they supply the body with essential supplements. The consumption of raw vegetables is the main way for transmission of intestinal parasitic organisms. This study was aimed at detecting the parasitic contamination in fresh vegetables sold in two central open-aired markets in Khartoum state, Sudan.

**Methods:**

In this prospective cross-sectional study, a total of 260 fresh vegetable samples and 50 water samples used to sprinkle vegetable(s) were collected from two central open-aired markets (namely; Elshaabi and Central markets) during November 2011 to May 2012. The samples were microscopically examined for detection of parasitic life forms using standardized parasitological techniques for protozoans and helminthes worms.

**Results:**

Of the 260 fresh vegetable samples, 35 (13.5 %) were microscopically positive for intestinal parasites whereas 7/50 (14 %) of water samples used to sprinkle vegetable(s) were found positives. Remarkably, high level of contamination in fresh vegetable samples was recorded in lettuce (*Lactuca sativa*) 36.4 % (4/11) while cayenne pepper (*Capsicum annuum*) and cucumber (*Cucumis sativus*) were not contaminated. The identified protozoans and helminthes were *Entamoeba histolytica/dispar*, *Entamoeba coli*, *Giardia lamblia*, *Ascaris lumbricoides*, *Strongyloides stercoralis*, *T. trichiura* and hookworms. The most predominant parasite encountered was *E. histolytica/dispar* (42.9 %) whereas both *T. trichiura* and *A. lumbricoides* (2.9 %) were the least detected parasites. None of the fresh vegetables had single parasitic contamination. The highest percentages found in water samples used to sprinkle vegetable(s) was for *Strongyloides* larvae 60 % (3/5). It is worth-mentioned that the rate of contamination in Elshaabi market was higher compared with Central market. However, there was no significant correlation between the type of vegetables and existence of parasites in both markets and a high significant relationship was observed between the type of parasite and total prevalence in fresh vegetables (p = 0.000).

**Conclusion:**

The study has identified a moderate rate of fresh vegetables contaminated with protozoan and helminthes. Contaminated fresh vegetables in central markets of Khartoum state may play a significant role in transmission of intestinal parasitic infections to humans, and the water used by greengrocers to sprinkle vegetable(s) can be implicated in vegetable contamination.

## Background

Vegetables are essential for a healthy human body, as they form a major component of human diet in every family [1]. Consuming fresh vegetables usually reduces the risk of stroke, cardiovascular diseases, and protects against certain types of cancers [2, 3]. Moreover, vegetables are vital sources of energy that are depended upon by all levels of human as food supplements or nutrients [4]. They substantially improve food quality as rich sources of water, vitamin C, carotene, mineral elements such as iron, and vitamins including thiamine (vitamin B12), niacin and riboflavin [5–7].

In many countries, vegetables are eaten raw or lightly cooked to preserve flavor, and this practice may favor the likelihood of food-borne parasitic infections [8, 9]. Vegetables become a potential source of human infections like enteric bacterial, viral and parasitic pathogens by contamination during production, collection, transport, preparation and/or during processing [2, 3, 10]. Additionally, the sources of contamination more often are soil, faeces (human and animal origin), water (irrigation, cleaning) [3, 5, 11]. More still, contamination may also occurs when fresh vegetables are rinsed and sprinkling with contaminated water [3, 12].

Recently, it has been reported that there is an increasing number of cases of food-borne illness mainly linked to eating fresh vegetables [2, 9, 12]. Parasitic infections lead to about 300 million severely illnesses with approximately 200,000 deaths occurring in developing countries [7]. Tremendous outbreaks of intestinal parasitic infections that were associated with raw vegetables have been reported from developed and developing countries as well [5, 10], these probably were due to poor sanitation and inadequate personal hygiene [13]. Several surveys have been done in different parts of the world such as in Syria [2]; Ghana [7]; India [9]; Pakistan [10]; Iran [5, 6, 12]; Nigeria [1, 3, 4, 14]; Vietnam [15, 16]; Ethiopia [11, 13, 17]; Egypt [8, 18] indicated that the vegetables can be a major source for transmitting protozoan cysts (*E. histolytica*; *Giardia lamblia*; *E. coli*; *Balantidium coli*), oocysts (*Isospora belli*; *Cryptosporidium* spp.) and helminthes’ eggs and larvae (*Strongyloides stercoralis*; *T. trichiura*; *Enterobius vermicularis*; *Fasciola hepatica*; *A. lumbricoides*; *Toxocara spp.*; *Hymenolepis nana*; *Hymenolepis diminuta*; *Taenia* spp.).

Notwithstanding, eating of raw vegetables and salads is the most common practice among Sudanese societies. Accordingly, the risk of intestinal parasitic infections increases especially when they are insufficiently washed. In that vein, the present study endeavors to detect parasites in fresh vegetables, because it is not enough to depend merely on the chemotherapeutic intervention of identified cases, but efforts needed to reduce and eliminate the potential sources of infection in Sudanese local populations. All of these have urged the undertaking of this study to investigate the level of parasitological contamination of fresh vegetables sold in central open-aired markets in Khartoum state, Sudan.

## Methods

### Study area

**Fig. 1:**
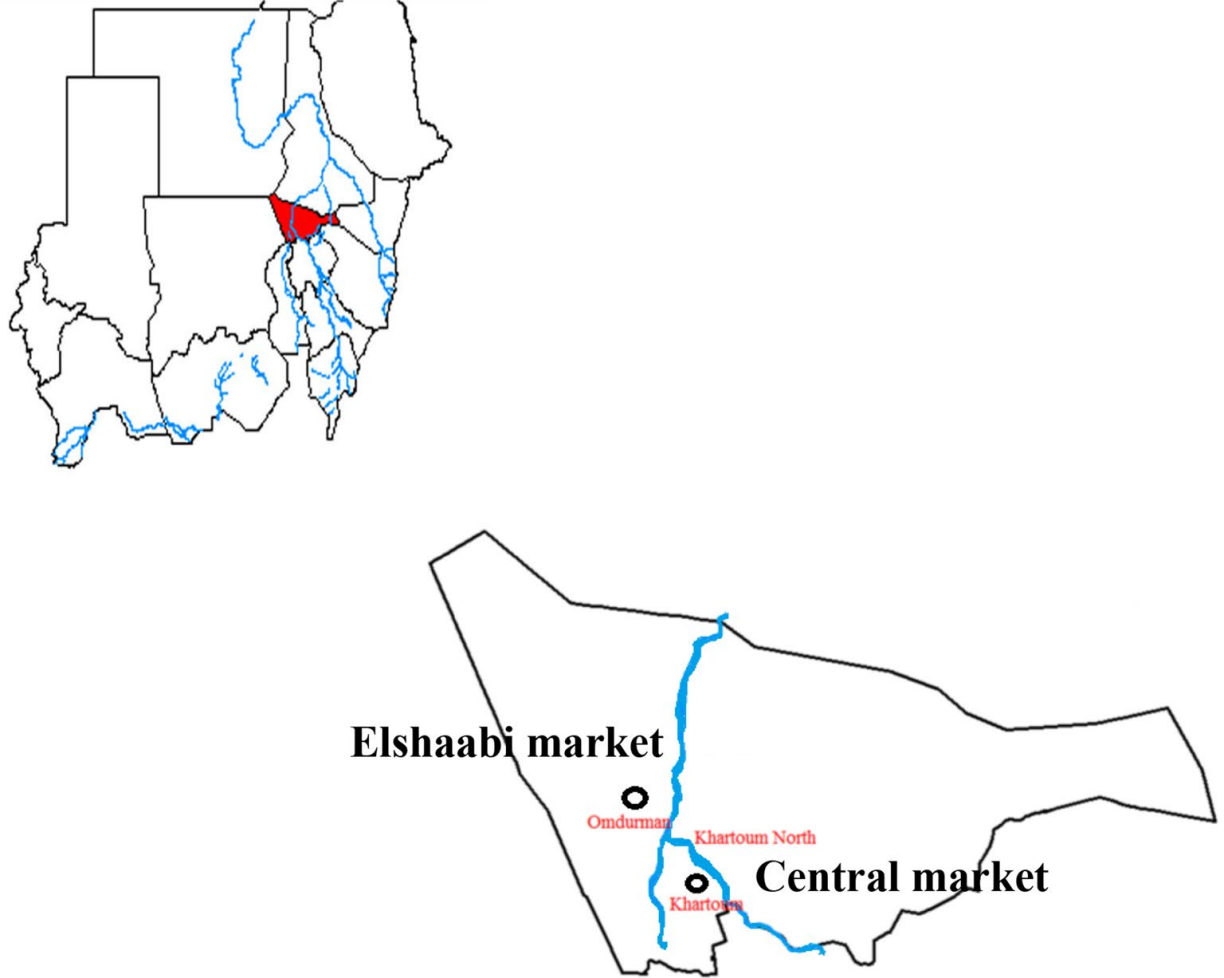
Map shows the study area (Elshaabi market in Omdurman and Central market in Khartoum)

A prospective cross-sectional study was conducted in Khartoum state during November 2011 to May 2012. Fresh vegetable samples were collected randomly from two central open-aired markets; Elshaabi market in Omdurman town and Central market in Khartoum town (Fig. 1). These markets were considered central as majority of fresh vegetables as well as fruits were brought from different farms and agricultural schemes in different parts of Sudan and sold in these markets. These vegetables were pre-washed before placed on shelves of markets.

### Sample collection and processing

A total of 260 fresh vegetable samples including 11 different types that are frequently consumed were picked randomly after getting consent to participate in the study. Vegetables were taken from greengrocers in the central open-aired markets (130 samples/market). In each market, samples were collected under normal purchase conditions from three randomly selected sellers. Samples were collected from the upper, middle and lower shelves of each seller. Likewise, fifty water samples used to sprinkle vegetable(s) were collected (25 samples/market).

The fresh vegetable samples collected in this study were tomatoes (*Solanum lycopersicum*), lettuce (*Lactuca sativa*), carrots (*Daucus carota*), cucumber (*Cucumis sativus*), Armenian cucumber (*Cucumis meloflexuosus*), radish (*Raphanus sativus*), watercress (*Nasturtium officinale*), green onion (*Allium cepa*), beet (*Beta vulgaris*), cayenne pepper (*Capsicum annuum*), and green pepper (*Capsicum* sp.). Fresh vegetables were collected into sterile, labeled polythene bags and transported immediately to the laboratory of Parasitology and Medical Entomology at Faculty of Medical Laboratory Sciences, University of Khartoum for parasitic examination. The label was referred to sample type, date of collection and name of market. Water samples used to sprinkle vegetable(s) were collected in clean universal containers (60 ml).

The fresh vegetable samples were washed in 10 % formal saline (150 ml) for detaching the parasitic stages (ova, larvae, cysts, and oocysts) of helminthes and protozoan parasites commonly assumed to be associated with vegetable contamination. The washing saline and water samples used to sprinkle vegetable(s) were transferred to 12 ml conical glass centrifuge tubes. For concentrating the parasitic stages, the tubes were centrifuged at 3000 rpm for 5 min [1]. After centrifugation, the supernatant was carefully siphoned off without shaking. Then the sediment was agitated gently by hand for redistributing the parasitic stages. Finally, the sediment was examined under light microscope using 10× and 40× objectives. Three slides were prepared from each sample to increase the chance of parasite detection. Then an iodine stained smear was prepared by adding a small drop of Lugol’s iodine solution prior to coverslipping to a slide similarly prepared for the unstained smear. The eggs/cysts were identified based on morphological details as described by Soulsby [19].

### Statistical analysis

Data analysis was done using SPSS 20.0. The Chi square test and analysis of variance ANOVA were used to find out the association between the acquisition of parasitic infection in fresh vegetables and type of parasites and to check the significance between the type of contaminated parasite and type of vegetable and water samples used to sprinkle vegetable(s). The p value <0.005 was considered as significant.

## Results

**Fig. 2:**
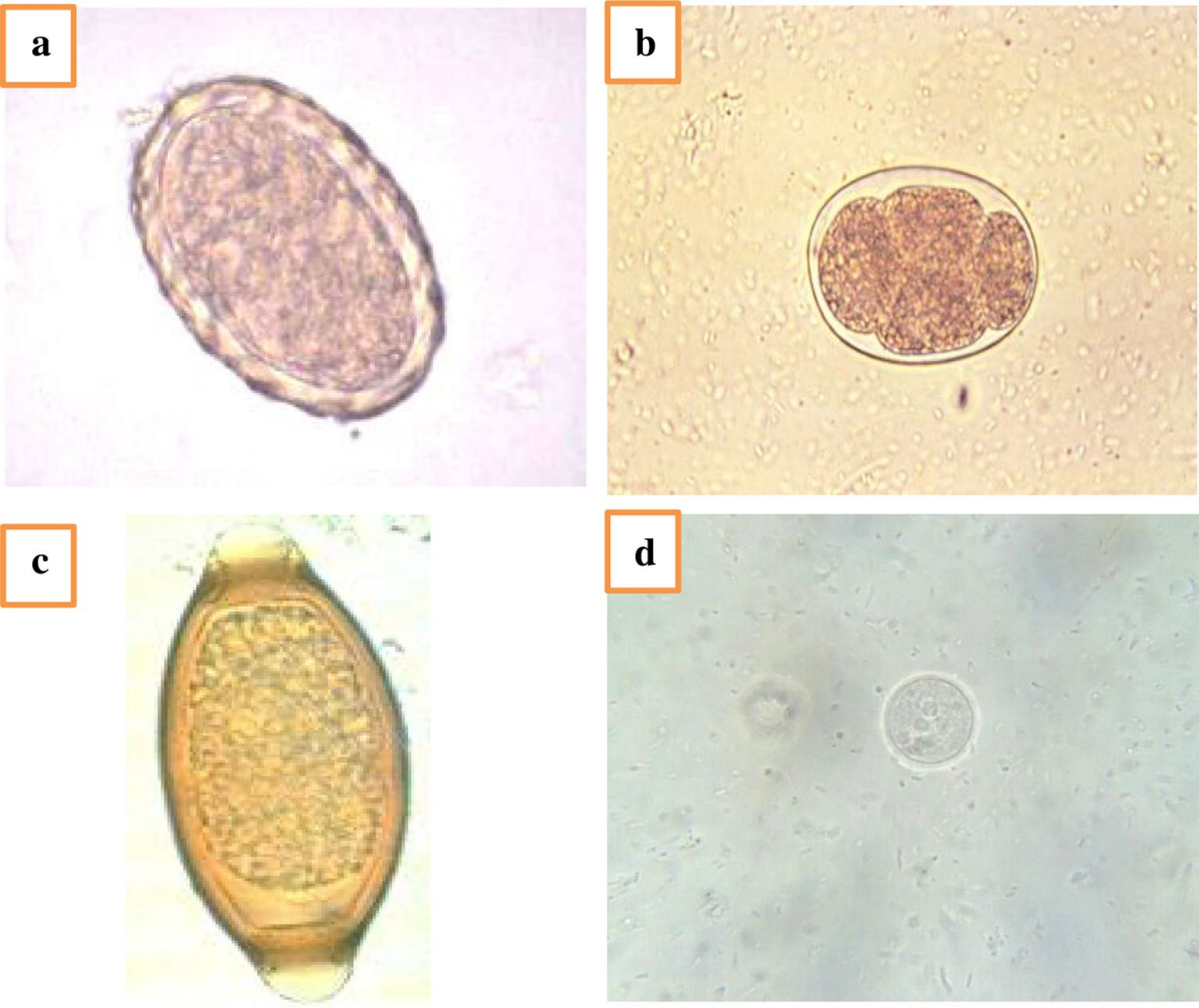
Intestinal helminths isolated in this study. **a** *A. lumbricoides* egg; **b** Hookworm egg; **c** *T. trichiura* egg; **d** *E. coli* cyst; (wet mount ×40)

A total of 260 fresh vegetable samples and 50 water samples used to sprinkle vegetable(s) were examined for the presence of parasite contamination. Helminthic eggs and protozoan cysts were detected in 13.5 % (35/260) of fresh vegetables examined and in 14 % (7/50) of water samples used to sprinkle vegetable(s) (Table 1). Interestingly, the most detected parasites in the vegetable samples in both markets were *E. histolytica/dispar* (42.9 %), *G. lamblia* (22.9 %), *E. coli* (14.3 %), *Strongyloides stercoralis* (8.6 %), hookworm’s eggs (5.7 %), *T. trichiura* and *A. lumbricoides* (2.9 %) for each (Table 2) (Fig. 2a, b). The most contaminated vegetables were lettuce (36.4 %) followed by watercress (30.4 %) and no parasite was detected in both of cucumber and cayenne pepper (0 %) (Table 1).

**Table 1:** Distribution of intestinal parasitic contamination in different fresh vegetables among the two markets

Vegetable type	No. of examined samples		*Central* market		*Elshaabi* market	
	Examined	Positive (%)	No. of examined	No. of positives (%)	No. of examined	No. of positives (%)
Tomatoes	36	4 (11.1)	21	2 (9.5)	15	2 (13.3)
Cucumber	12	0 (0)	4	0 (0)	8	0 (0)
Armenian cucumber	16	3 (18.8)	5	0 (0)	11	3 (27.3)
Green pepper	25	2 (8)	14	2 (14.3)	11	0 (0)
Cayenne pepper	7	0 (0)	3	0 (0)	4	0 (0)
Radish	24	2 (8.3)	16	2 (12.5)	9	0 (0)
Beet	19	3 (15.8)	11	1 (9.1)	8	2 (25)
Watercress	23	7 (30.4)	14	5 (35.7)	9	2 (22.2)
Lettuce	11	4 (36.4)	5	1 (20)	6	3 (50)
Green onion	36	5 (13.9)	18	2 (11.1)	18	3 (16.7)
Carrot	50	5 (10)	19	0 (0)	31	5 (16.1)
Total	260	35 (13.5)	130	15 (11.6)	130	20 (15.4)
Vegetables’ refreshing water	50	7 (14)	25	3 (12)	25	4 (16)
Total	50	7 (14)	25	3 (12)	25	4 (16)

**Table 2:** Distribution of intestinal parasitic contamination in fresh vegetable samples and vegetables’ refreshing water samples among the two markets

Detected organism	Total prevalence in fresh vegetables (%)	Total prevalence in vegetables’ refreshing water (%)
*E.* spp. cyst	15 (42.9)	–
*G. lamblia* cyst	8 (22.9)	2 (28.6)
*E. coli* cyst	5 (14.3)	1 (14.3)
*S. stercoralis* ova	3 (8.6)	3 (42.9)
Hookworms egg	2 (5.7)	–
*T. trichiura* ova	1 (2.9)	–
*A. lumbricoides* ova	1 (2.9)	2 (28.6)

The predominant parasites found in water samples used to sprinkle vegetable(s) were *Strongyloides stercoralis* (42.9 %), *G. lamblia*, *A. lumbricoides* (28.6 %) for each, and finally *E. coli* (14.3 %) (Table 2).

Furthermore, the rate of contamination among fresh vegetables examined in Elshaabi market was much higher (13.8 %) compared with Central market (10.8 %) (Table 1). Similarly, in water samples used to sprinkle vegetable(s) in Elshaabi market was high (15.4 %) compared with Central market (11.6 %) (Table 1).

It is worth mentioning that among all examined fresh vegetable samples none of them had single parasitic contamination (Table 3) and there was multiple contamination (*G. lamblia* and *A. lumbricoides*) recorded in single water sample used to sprinkle vegetable(s) collected from Central market (Table 2).

**Table 3:** Distribution of intestinal parasites in relation to the type of fresh vegetable samples collected from both markets

Vegetables	*E. coli* cyst	*E.* spp. cyst	*G. lamblia* cyst	*A. lumbricoides* ova	*S. stercoralis* ova	Hookworms egg	*T. trichiura* ova	Total
Tomatoes	1	1	1	1	0	0	0	4/36
Cucumber	0	0	0	0	0	0	0	0/12
Armenian cucumber	0	2	0	0	0	0	1	3/16
Green pepper	0	1	1	0	0	0	0	2/25
Cayenne pepper	0	0	0	0	0	0	0	0/7
Radish	1	0	1	0	0	0	0	2/24
Beet	1	1	0	0	0	1	0	3/19
Watercress	1	1	2	0	2	1	0	7/23
Lettuce	1	2	1	0	0	0	0	4/11
Green onion	0	4	1	0	0	0	0	5/36
Carrot	0	3	1	0	1	0	0	5/50
Total	5	15	8	1	3	2	1	35/260

However, there was no significant correlation between the type of vegetable and existence of parasites in both markets (p < 0.301). Importantly, a significant relationship was observed between the type of parasite and total prevalence in fresh vegetables (p < 0.000) in both markets.

**Fig. 3:**
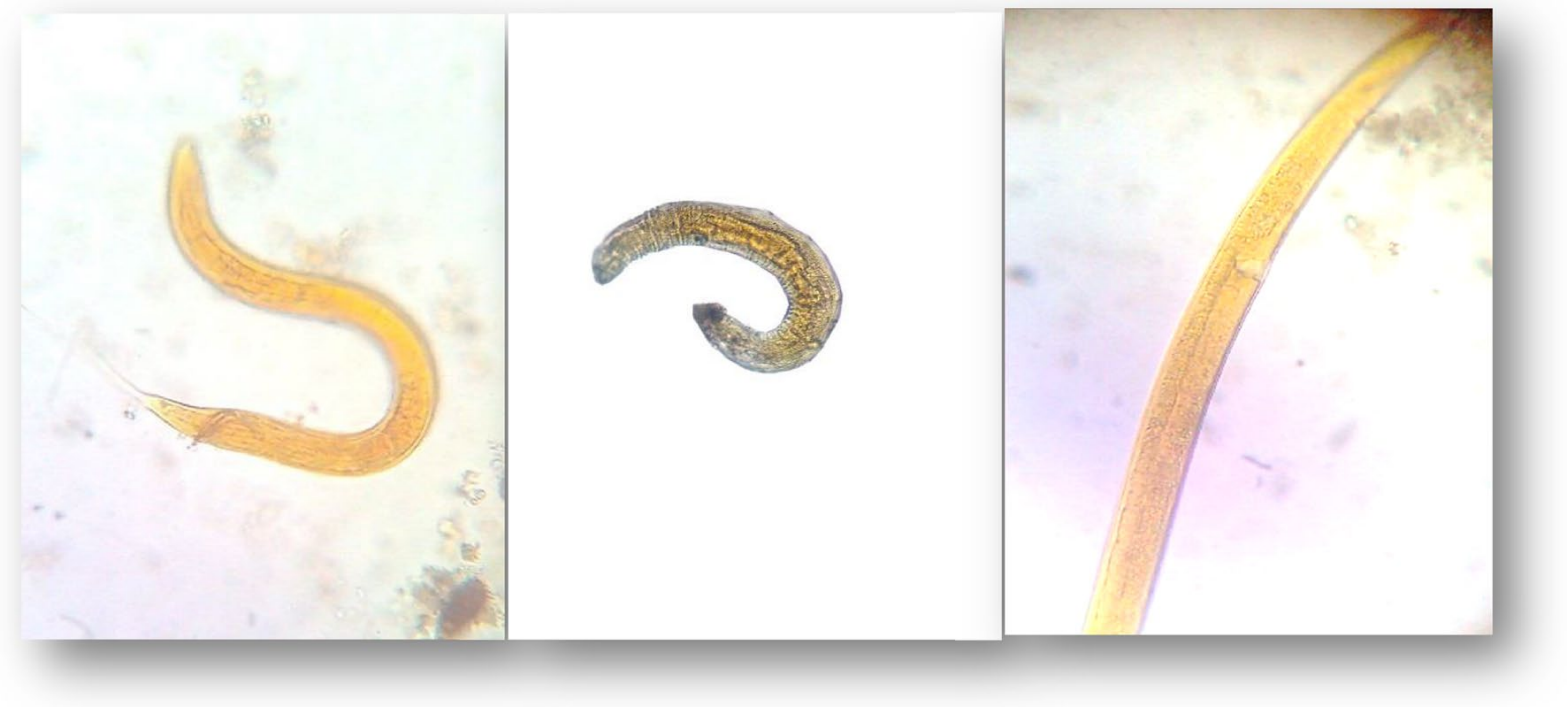
Different non-pathogenic nematode larvae and adults (×40)

In this study larvae and adult nematodes were detected among around 34 samples of fresh vegetables. The largest numbers were detected in green onion and beet, but these stages were excluded because of their characteristic features that differ from that of human pathogens (Fig. 3).

## Discussion

The force of habit of eating raw vegetables plays a critical epidemiological role in transmitting parasitic food-borne diseases [2, 6]. Recovery of parasites from fresh sold vegetables is an indication of the quality of the overall process of cultivation, irrigation and post-harvest handling, and may be helpful in indicating the incidence of intestinal parasites among a local community [2, 3].

The present study has shown that 11 fresh vegetables routinely used for human consumption in Khartoum state, Sudan, were contaminated with several parasitic stages. Furthermore, study by Shahonazi and Jafari-Sabet 2010 [20] has pointed out that the poorly washed vegetables are considered to be a major route for transmitting intestinal parasitic infections. Unfortunately, the prevalence rate of contaminated vegetable samples was found to be 13.5 % whereas for water samples used to sprinkle vegetable(s) was found to be 14 % in both markets. Fortunately, comparing this result with other studies, it was lower than many studies done in Syria [2], Iran [5, 6, 12], Pakistan [10], Ethiopia [11, 13, 17], Egypt [8, 18], Nigeria [1, 3, 4] and Vietnam [16]. Additionally, it is much higher than a study has been conducted in Nigeria [14] with 11.0 % prevalence. Probably, this variation in contaminations may be attributed to geographical location, type and number of samples examined, methods used for detection, different laboratory techniques used, type of water used for irrigation, post harvesting handling methods of such vegetables and even the type of water used to wash vegetables can play an instrumental role in the epidemiology of transmission of parasitic diseases.

The present study was in assent with many exhaustive studies [2, 9, 10, 12, 18] in that the lettuce had the highest prevalence of contamination, and this may be ascribed to it is leaves which are capable of harboring parasites in-between and in addition to its uneven surfaces on which parasites are attached more easily than other vegetables with smooth surfaces i.e. cucumber, cayenne pepper, green pepper and tomatoes and this observation in accordance with finding by Damen (2007) [21].

It is well evident that the most detected parasite was *E. histolytica/dispar* in fresh vegetable samples, and this result in accordance with study conducted by Benti and Gemechu (2014) [17]. Notwithstanding, cayenne pepper and cucumber were found free of parasites and this in accord with ul-Hag et al. (2014) [10] who found that cayenne pepper was less contaminated by parasitic infections in Lahore, Pakistan.

Post-harvest faecal contamination which may occurs during handling and transport usually occurs through splashing the vegetables with contaminated water from dirty containers or unhygienic handling in order to keep them fresh. This is supported by this study in which there was 14 % of 50 water samples used to sprinkle vegetable(s) were found to harbor parasitic stages. The greengrocers of the markets buy water for washing and freshening vegetables. The water is brought from outside the market in containers carried by cart because the markets lack healthy piped water form reservoir. Most of the water samples used to sprinkle vegetable(s) were dirty due to dust and vegetable debris.

All isolated fresh vegetable samples and one sample of water used to sprinkle vegetable(s) had more than one species of parasite per sample, and this reflects the magnitude of a single faecal contamination of vegetables which may results in several parasitic infections [14].

## Conclusions

In conclusion, few types of fresh vegetables in Khartoum area were moderately contaminated by intestinal parasites than other areas. This is suggested that humans are at high risk of getting infections from contaminated fresh vegetables eaten on daily basis. These findings raised concern of public health being at high risk of infection with amoebiasis, G.sis, strongyloidiasis, ascariasis and others. So, adopting control measures that cover guidelines of irrigation water quality, strategies to reduce the risk of disease transmission by food-borne parasites, preventing domestic and wild animals from entering into the plant farms and avoiding to use untreated night soil as fertilizer is highly recommended. Effective and comprehensive prevention and treatment measures should be taken to ensure food safety. Washing procedures before eating raw vegetables regardless of the provider’s sanitation should be performed to avoid transmission of intestinal parasites.
